# Cationic Antimicrobial Peptides Inactivate Shiga Toxin-Encoding Bacteriophages

**DOI:** 10.3389/fchem.2017.00122

**Published:** 2017-12-19

**Authors:** Manuel E. Del Cogliano, Axel Hollmann, Melina Martinez, Liliana Semorile, Pablo D. Ghiringhelli, Paulo C. Maffía, Leticia V. Bentancor

**Affiliations:** ^1^Laboratory of Genetic Engineering and Molecular Biology, Institute of Basic and Applied Microbiology, National University of Quilmes, Bernal, Argentina; ^2^Consejo Nacional de Investigaciones Científicas y Técnicas, Buenos Aires, Argentina; ^3^Laboratory of Biointerfaces and Biomimetic Systems, CITSE, National University of Santiago del Estero, Santiago del Estero, Argentina; ^4^Laboratory of Molecular Microbiology, Institute of Basic and Applied Microbiology, National University of Quilmes, Bernal, Argentina

**Keywords:** bacteriophages (phages), antimicrobial peptides, *Escherichia coli* O157, hemolytic uremic syndrome (HUS), anti-infective agents

## Abstract

Shiga toxin (Stx) is the principal virulence factor during Shiga toxin-producing *Escherichia coli* (STEC) infections. We have previously reported the inactivation of bacteriophage encoding Stx after treatment with chitosan, a linear polysaccharide polymer with cationic properties. Cationic antimicrobial peptides (cAMPs) are short linear aminoacidic sequences, with a positive net charge, which display bactericidal or bacteriostatic activity against a wide range of bacterial species. They are promising novel antibiotics since they have shown bactericidal effects against multiresistant bacteria. To evaluate whether cationic properties are responsible for bacteriophage inactivation, we tested seven cationic peptides with proven antimicrobial activity as anti-bacteriophage agents, and one random sequence cationic peptide with no antimicrobial activity as a control. We observed bacteriophage inactivation after incubation with five cAMPs, but no inactivating activity was observed with the random sequence cationic peptide or with the non-alpha helical cAMP Omiganan. Finally, to confirm peptide-bacteriophage interaction, zeta potential was analyzed by following changes on bacteriophage surface charges after peptide incubation. According to our results we could propose that: (1) direct interaction of peptides with phage is a necessary step for bacteriophage inactivation, (2) cationic properties are necessary but not sufficient for bacteriophage inactivation, and (3) inactivation by cationic peptides could be sequence (or structure) specific. Overall our data suggest that these peptides could be considered a new family of molecules potentially useful to decrease bacteriophage replication and Stx expression.

## Introduction

Infections with Shiga toxin-producing *Escherichia coli* (STEC) strains are a serious public health problem. Children infected with STEC strains present diarrhea, hemorrhagic colitis, and a percentage of patients can develop Hemolytic Uremic Syndrome (HUS).

Shiga toxin (Stx) is the main virulence factor during STEC infections. Because the gene encoding for Stx is inside the prophage genome, these strains are also known as *Escherichia coli* Shiga Toxin-Encoding Bacteriophages. During STEC infection, the bacteriophage is cleaved and the replication and Stx expression take place inside the gut. Then, free bacteriophages are able to infect other susceptible bacteria present in the gut, exacerbating Stx expression (Cornick et al., 2006). Currently, there are no effective treatments or vaccines available, and for this reason bacteriophage inactivation treatments are a promising strategy to prevent Stx expression after STEC infections.

Previously, we showed that chitosan has anti-bacteriophage activity *in vitro* and *in vivo* (Amorim et al., 2014). Chitosan is a cationic linear polysaccharide polymer obtained after the deacetylation of chitin; this polymer has been widely-used as an antimicrobial agent against several microorganisms (Kong et al., 2010). In a previous work Maffía and collaborators designed a group of new cationic antimicrobial peptides and tested them against a broad panel of multi-resistant clinical bacterial isolates (Faccone et al., 2014). In order to evaluate whether these sequences could affect bacteriophage infection we tested seven previously designed cAMPs (Faccone et al., 2014; Hollmann et al., 2016; Maturana et al., 2017) as potential anti-bacteriophage agents. To analyze the effect of these peptides, we used them to inactivate a previously reported mutant bacteriophage (ϕΔTOX:GFP) in which the *stx* operon has been replaced by a gene encoding for the green fluorescent protein (GFP) (Amorim et al., 2014). Five of these peptides were previously analyzed and displayed antimicrobial activity in different bacterial strains and structure as alpha helix in contact with lipid membranes (P5, P8, P8.1, P2, and P6.2). The other two peptides tested were Omiganan, a linear Beta-sheet cAMP derived from indolicidin that underwent clinical trials with activity against *S. aureus*; and a random sequence peptide with cationic charge but no antimicrobial activity (Faccone et al., 2014; Hollmann et al., 2016).

Therefore, the objective of this work was to evaluate if this group of cAMPs could inactivate Shiga toxin- encoding bacteriophages.

## Materials and methods

### Cationic peptides

Each peptide was synthesized with C terminus amidation. Peptides were synthesized and obtained at a purity grade of >95% by HPLC (GenScript Co., Piscataway, NJ 08854, USA). Cationic alpha helical peptides P5, P8, P8.1, P2, and P6.2 were previously designed using a combined rational and computer assisted approach, identifying short putative active regions from AMP databases (Faccone et al., 2014; Maturana et al., 2017). Peptide sequences are: peptide 2: GLLKKWLKKWKEFKRIVGY; peptide 8.1: RIVQRIAKWAKKWYKAGK, peptide 6.2: GLLRKWGKKWKEFLRRVWK; peptide 5: RIVQRIKKWLLKWKKLGY; peptide 8: RIVQRILKWLKKWYKLGK. Omiganan (MBI-226): ILRWPWWPWRRK; Random non-alpha helical peptide: MVVFSVPKFKSTVAKLLSSA.

### Bacteriophage induction

*E. coli* C600ΔTOX:GFP strain was obtained from Dr. Weiss, University of Cincinnati (Gamage et al., 2003). *E. coli* C600ΔTOX:GFP is a lysogenized C600 strain carrying the 933W bacteriophage in which the stx gene was replaced by the gfp sequence. Since this strain does not produce Shiga toxin, it represents a safety option to evaluate bacteriophage infection. *E. coli* C600ΔTOX:GFP strain was grown in Luria Broth (LB) plus 10 mM CaCl_2_ and chloramphenicol (Sigma) (15 μg/ml final concentration) overnight (ON) at 37°C under agitation. The ON culture was diluted to OD 600 nm = 0.1 in LB plus 10 mM CaCl_2_ and chloramphenicol (Sigma) (15 μg/ml final concentration). Induction was carried out by adding ciprofloxacin to a final concentration of 40 ng/ml (Ciprax 200, Roemmers). Bacteria were incubated for 6 h at 37°C under agitation and cultures were then centrifuged at 5,000 rpm for 15 min. The bacteriophage-containing supernatant was purified by sucrose ultracentrifugation. Briefly, supernatant containing bacteriophage was ultracentrifugated at 35.000 × g (Beckman XL-70, Rotor J-20), at 4°C during 2 h with 35% sucrose solution. The pellet was resuspended in 1 ml of PBS, filtered with 0.2 μm filters (MC-PES-02S, Microclar) and kept at 4°C until the titration assay was performed.

### Titration assay

*E. coli* strain (ATCC 37197) was grown in LB plus ampicillin (0.05 mg/ml final concentration) overnight at 37°C under agitation at 200 rpm. The culture was diluted 1:100 in LB plus ampicillin (0.05 mg/ml final concentration) and incubated for 2 additional hours at 37°C under agitation. At the end of the incubation, 1,000 μl samples of the *E. coli* strain were incubated with 100 μl of a suspension containing bacteriophages for 30 min at room temperature. At the end of this incubation, 3 ml of Top Agar (Tryptone 1%; NaCl 0.5%; Agar 0.7%) plus CaCl_2_ (10 mM final concentration) was added, and plated on LB-Amp agar plates. Plates were incubated at 37°C and lysis plaques were counted after 24 h.

### Anti-bacteriophage activity

Bacteriophage ΔTOX:GFP (ϕΔTOX:GFP) was incubated with peptides diluted in 400 μl of PBS at a final concentration of 0.1 μg/ml, 5 μg/ml, 10 μg/ml, or 50 μg /ml for 16 h at 37°C. After incubation, bacteriophage titers were measured as described above. *E. coli* strain (ATCC 37197), used for titration assay, was incubated with peptides alone as a control. During titration assay, the final concentration of peptides in the bacterial suspension was 1.4, 2.8, and 14 μg/ml, respectively.

### Z potential

The Zeta potential measured in volts (ξ) was calculated using the Smoluchowski equation: where η is the viscosity of the suspension at 20°C, D is the dielectric constant of the solution at 20°C and μ is the electrophoretic mobility of particles (micrometer/s per volt/cm). Bacteriophage was incubated with a solution containing peptide 5 or Omiganan at 5.7 μg/ml final concentration for 30 min at 25°C under gentle agitation. Measurements were conducted in a Nano Particle Analyzer SZ100 (Horiba) at 25°C, each value represents the average of two independents batches, and 100 individual determinations were obtained per batch.

### Dynamic light scattering

Dynamic light scattering experiments were carried out on an Analyzer SZ100 (Horiba) with a backscattering detection at 173°, using disposable polystyrene cells. The bacteriophage suspensions (with or without peptide) were left equilibrating for 15 min at 25°C. Normalized intensity autocorrelation functions were analyzed using the CONTIN method (Provencher, 1982), yielding a distribution of diffusion coefficients (*D*). *D* is used to calculate the hydrodynamic diameter (*D*_*H*_) through the Stokes-Einstein relationship: (3) where *k* is the Boltzmann constant, T the absolute temperature, and η the viscosity of the medium. The *D*_*H*_ value was calculated from a set of 15 measurements (~13 runs each) for the bacteriophage in presence of peptide p5 or alone. The *D*_*H*_ of the sample was obtained from the peak with the highest scattered light intensity (i.e., the mode) in light scattering intensity distributions.

### Statistical analysis

The significance of the difference between concentrations was analyzed using Prism 5.0 software (GraphPad Software), and the *P*-value is indicated by asterisks in the figures. Data correspond to mean ± standard errors of the mean (SEM) for each concentration using triplicates. Statistical differences were determined using the one-way analysis of variance (ANOVA). Comparisons a posteriori between groups were performed using Tukey's Multiple Comparison Test analysis.

## Results

### Anti-bacteriophage activity

**Figure 1:**
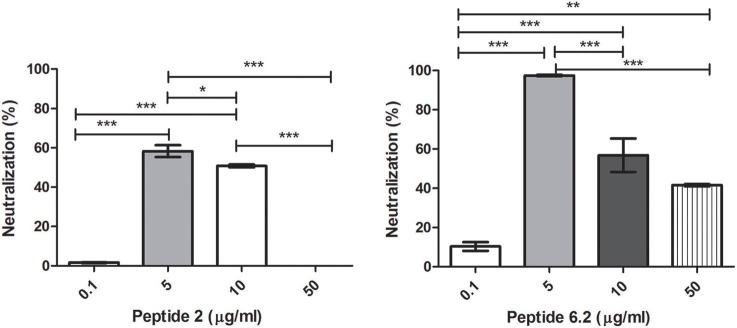
Bacteriophage inactivation by cationic peptides. **(A)** Peptide P.2. **(B)** Peptide 6.2. After pre-incubation of bacteriophages with different concentrations of peptides the inactivation was measured by titration of *E. coli*, strain (ATCC 37197). Prism 5.0 software (GraphPad Software) was used to determine statistical significance between different samples. Peptide 2: ^*^p < 0.05, ^***^p < 0.0001. Peptide 6.2: ^**^p < 0.05, ^***^p < 0.0001.

**Figure 2:**
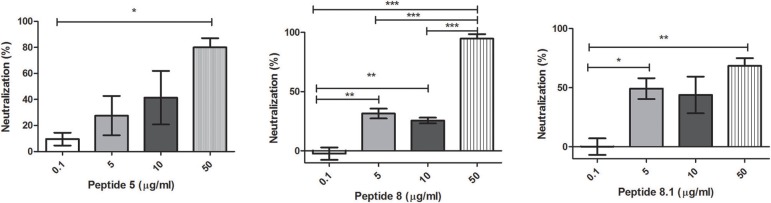
Bacteriophage inactivation by cationic peptides. **(A)** Peptide 5 **(B)** Peptide 8 **(C)** Peptide 8.1. After pre-incubation of bacteriophages with different concentrations of peptides the inactivation was measured by titration of *E. coli*, strain (ATCC 37197). Prism 5.0 software (GraphPad Software) was used to determine statistical significance between different samples using one-way analysis of variance (ANOVA). Peptide 5: ^*^p = 0.0313. Peptide 8: ^**^p < 0.005, ^***^p < 0.0001. Peptide 8.1: ^*^p < 0.05, ^**^p = 0.008.

Bacteriophages were incubated with 0.1, 5, 10, or 50 μg/ml of each peptide. After incubation, inactivation was evaluated analyzing the bacteriophage capacity to infect *E. coli*. The seven peptides displayed different inactivation activities against the bacteriophage. Peptides 6.2, 5, and 8 showed the highest inactivation activity, inactivating nearly the 100% of phages in the conditions tested (Figures 1B, 2A,B).

On the other hand, Peptides 2 and 8.1 showed the lowest inactivation activity among the cAMP tested (Figures 1A, 2C).

**Figure 3:**
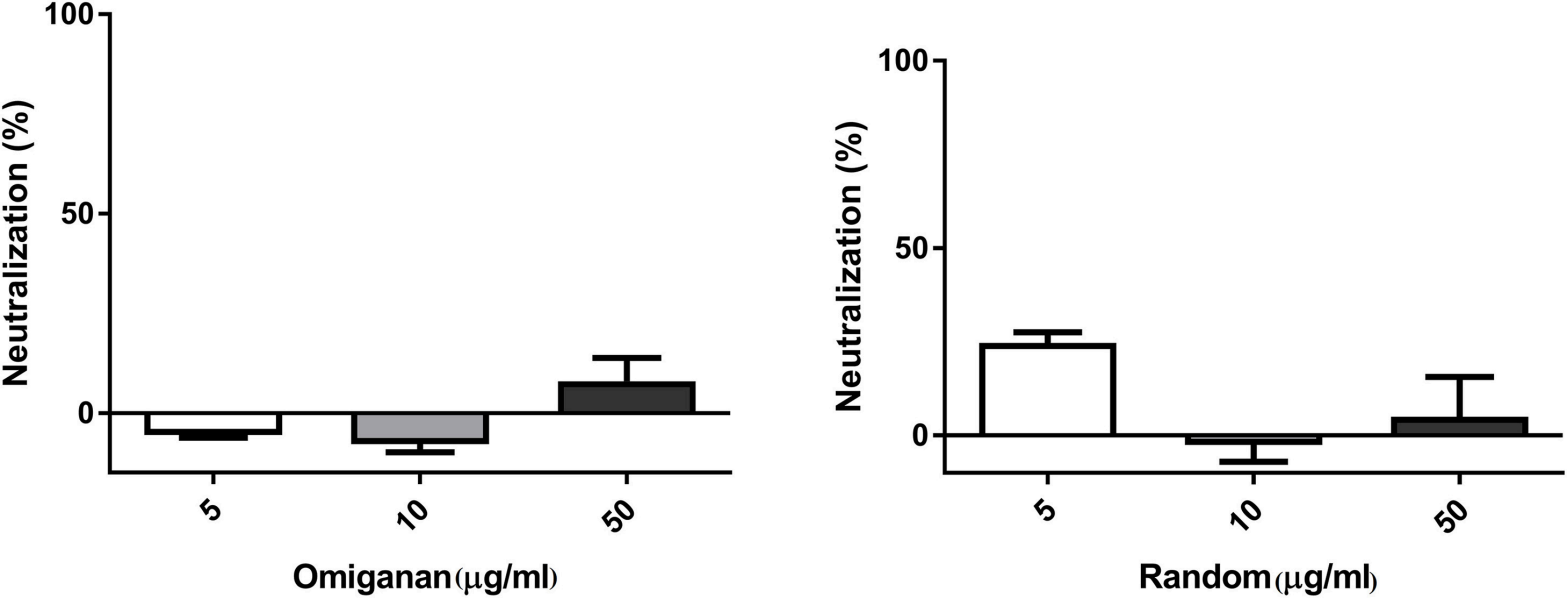
Bacteriophage inactivation by control peptides. **(A)** Omiganan, a commercial cationic beta sheet peptide **(B)** Random sequence, a cationic non alpha helical peptide. Prism 5.0 software (GraphPad Software) was used to determine statistical significance between different samples using one-way analysis of variance (ANOVA). Omiganan: ns. Random: ns.

Random peptide and Omiganan showed no inactivation activity against bacteriophage under the conditions tested (Figure 3).

It is important to notice that, according to the protocol we used, after the incubation of each peptide with phages, the peptide-phage mixture is plated on the *E. coli* lawn. For that reason a possible antimicrobial activity of these cAMPS on this *E. coli* strain had to be previously assessed, and no visible activity of the peptides *per se* was observed. It is worth mentioning that the final peptide concentration affecting the bacterial lawn during the titration assay ranged from1.4 to 14 μg/ml, which is a much lower concentration than the effective minimal inhibitory activity (MIC) previously obtained for each peptide on *E. coli*. In addition we performed a MIC assay for these peptides at different concentrations between 54 and 3.3 μg/ml on the *E. coli* C600ΔTOX:GFP strain, and we found no antimicrobial activity below 27 μg/ml for all the peptides tested.

### Bacteriophage-peptide interaction

**Figure 4:**
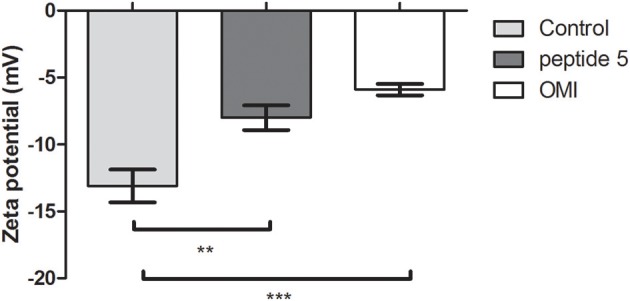
Zeta potential measurements of bacteriophage incubated with peptides P5 or Omiganan (OMI), or in buffer solution (control). Each point represents the mean of 2 independent batches ± SEM. ^**^P < 0.01; ^***^P < 0.001, one-way ANNOVA followed by a Dunnett post-test for multiple comparisons vs. the control.

The interaction between peptides and bacteriophages was analyzed using Zeta potential assays. We observed that peptide 5 was able to modify the surface charge of the bacteriophages, by decreasing the net negatively charge exposed by the bacteriophage. The results strongly suggest that positively charged peptides and bacteriophages are interacting through electrostatic charges (Figure 4). Interestingly, the same behavior was found with peptide Omiganan, although this peptide did not show anti-bacteriophage activity.

**Figure 5:**
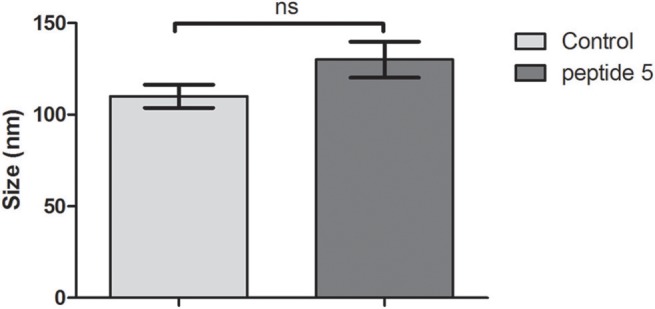
Average hydrodynamic diameter of bacteriophage incubated with peptides 5 or in buffer solution (control). Ns, Not significant, *t*-test (unpaired).

We used DLS to assess whether the size of the bacteriophage was altered as a consequence of its interaction with the peptides, or inducing any aggregation effect. The results suggest that peptides do not affect the hydrodynamic diameter of the bacteriophage (Figure 5).

## Discussion

It is well known that bacteriophage induction is required for HUS development (Tyler et al., 2013). Additionally, it was also reported that bacteriophage was able to infect bacteria *in vivo* and *in vitro* (Schmidt, 2001; Cornick et al., 2006).

We have previously observed a dramatical decrease of GFP expression *in vivo* in mice treated with chitosan after infection with a non-pathogenic *E. coli* strain containing a mutant bacteriophage in which the gene of *stx* was replaced by *gfp* sequence (Amorim et al., 2014).

In order to evaluate another anti-bacteriophage agent, we analyzed a group of cationic peptides. In this work, and for the first time ever reported, we observed that seven cAMPs were able to inactivate bacteriophage infection on bacteria. It is worth mentioning that in previous works we observed that all the peptides, except random peptide, displayed antimicrobial activity (Faccone et al., 2014; Hollmann et al., 2016; Maturana et al., 2017). Interestingly, even though they are both cationic sequences, Omiganan and random peptide did not display any bacteriophage inactivation activity. In regard to the structure, unlike the rest of the sequences tested, these two peptides did not structure as alpha helix in contact with lipidic membranes (Faccone et al., 2014).

It is interesting to note that for peptides 2 and 6.2 we observed a bell curve shaped behavior of the phage neutralizing activity vs. concentration curve. This kind of behavior is more or less common among some drugs, and in this case we could speculate that the tendency to aggregate that these particular peptides have at high doses, due to the high number of hydrophobic amino acids they harbor, could be responsible, at least in part, for this phenomenon. This tendency to aggregate, which depends on the amino acid composition and the structure the peptide sequence displays, is probably responsible for the inactivation of the peptide as we increase the concentration and more aggregates are formed.

In order to evaluate if a direct interaction between peptides and bacteriophage is involved in the inactivation activity, zeta potential experiments were conducted, using peptide 5 as a model. The result obtained (Figure 4) confirmed an electrostatic interaction between peptide and bacteriophage. However, Omiganan, that displayed no inactivation activity, also showed direct interaction with the bacteriophage. These findings allow us to hypothesize that cationic properties, that are probably responsible for the interaction between peptides and bacteriophage, are necessary but not sufficient for achieving an inactivation activity.

**Figure 6:**
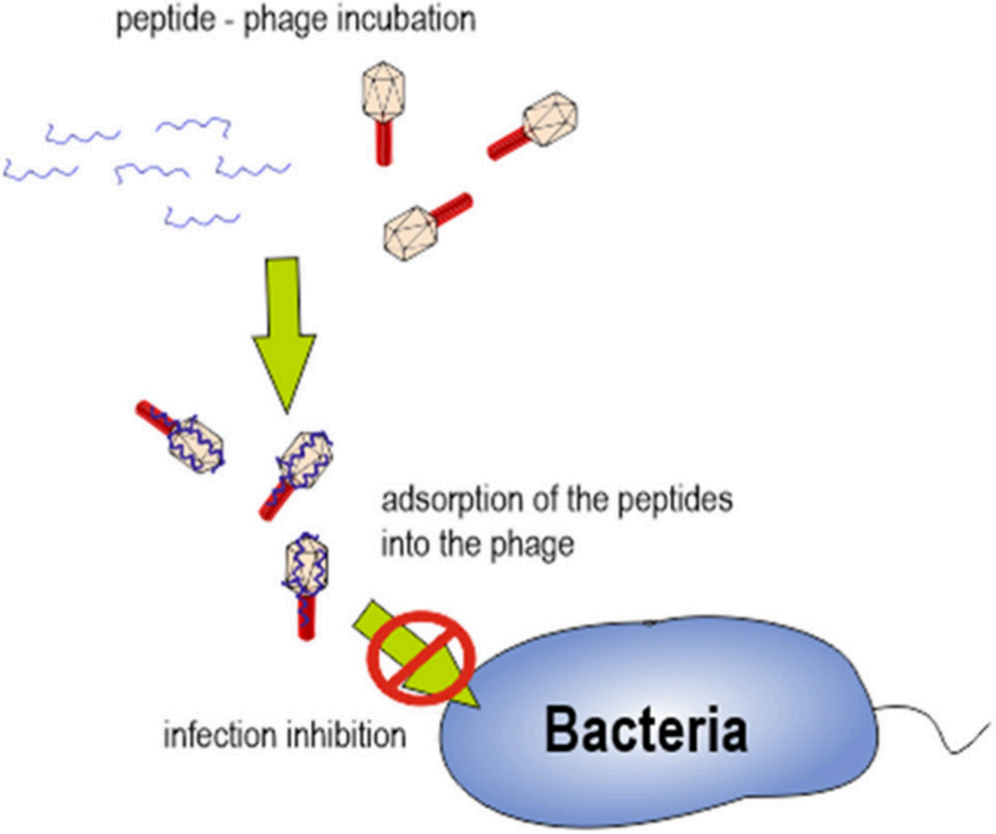
Schematic representation of bacteriophage-peptide interaction and its role in bacterial infection.

Overall, our results lead us to hypothesize that specific interaction between cAMPs and bacteriophage proteins, after a first approach driven by electrostatic force, might be responsible for infection inhibition. We hypothesize that the complex generated after bacteriophage-peptide incubation could be responsible for preventing the bacteriophage adhesion on the bacterial cell wall (Figure 6). Further experiments analyzing bacteriophage proteins implicated on bacterial adhesion should be performed.

In the race to find a therapy to decrease the risk of HUS development, bacteriophage inactivation by cAMPs could be a promising new strategy for the inhibition of the bacteriophage replication and Stx expression. These peptides could be considered a new family of molecules potentially useful for a future HUS treatment.

The results obtained in this work, using novel designed cAMPS, open another relevant area of study related with the interactions between natural immune cAMPs and bacteriophages, for instance cAMPS produced by human leukocytes at the site of infection. If these results could be replicated with human cAMPs, a whole new perspective of SUH development could arise, in which human cationic peptides could play a crucial role in the natural control of phage replication. Altogether, these results highlight that cationic peptides are potential candidates for future research in alternative treatments for STEC infections.

## Author contributions

LB designed, analyzed data and wrote the manuscript; PM provided advice on experimental design, analyzed data and wrote the manuscript; MD performed experiments and provided advice on experimental design, LS and PG provided substantial comments on the manuscript and on experimental design. MM and AH performed experiments and editing on the manuscript.

### Conflict of interest statement

The authors declare that the research was conducted in the absence of any commercial or financial relationships that could be construed as a potential conflict of interest.
